# Left atrial appendage size is a marker of atrial fibrillation recurrence after radiofrequency catheter ablation in patients with persistent atrial fibrillation

**DOI:** 10.1002/clc.23748

**Published:** 2021-11-19

**Authors:** Judit Simon, Mohammed El Mahdiui, Jeff M. Smit, Lili Száraz, Alexander R. van Rosendael, Szilvia Herczeg, Emese Zsarnóczay, Anikó Ilona Nagy, Márton Kolossváry, Bálint Szilveszter, Nándor Szegedi, Klaudia Vivien Nagy, Tamás Tahin, László Gellér, Rob J. van der Geest, Jeroen J. Bax, Pál Maurovich‐Horvat, Béla Merkely

**Affiliations:** ^1^ MTA‐SE Cardiovascular Imaging Research Group, Heart and Vascular Center Semmelweis University Budapest Hungary; ^2^ Medical Imaging Centre Semmelweis University Budapest Hungary; ^3^ Department of Cardiology Leiden University Medical Center Leiden The Netherlands; ^4^ Heat and Vascular Center Semmelweis University Budapest Hungary; ^5^ Division of Image Processing Leiden University Medical Center Leiden The Netherlands; ^6^ Heart Center Turku University Hospital and University of Turku Turku Finland

**Keywords:** atrial fibrillation, echocardiography, heart atria, recurrence, transesophageal, transthoracal, tomography, X‐ray

## Abstract

**Introduction:**

There are no consistently confirmed predictors of atrial fibrillation (AF) recurrence after catheter ablation. Therefore, we aimed to study whether left atrial appendage volume (LAAV) and function influence the long‐term recurrence of AF after catheter ablation, depending on AF type.

**Methods:**

AF patients who underwent point‐by‐point radiofrequency catheter ablation after cardiac computed tomography (CT) were included in this analysis. LAAV and LAA orifice area were measured by CT. Uni‐ and multivariable Cox proportional hazard regression models were performed to determine the predictors of AF recurrence.

**Results:**

In total, 561 AF patients (61.9 ± 10.2 years, 34.9% females) were included in the study. Recurrence of AF was detected in 40.8% of the cases (34.6% in patients with paroxysmal and 53.5% in those with persistent AF) with a median recurrence‐free time of 22.7 (9.3–43.1) months. Patients with persistent AF had significantly higher body surface area‐indexed LAV, LAAV, and LAA orifice area and lower LAA flow velocity, than those with paroxysmal AF. After adjustment left ventricular ejection fraction (LVEF) <50% (HR = 2.17; 95% CI = 1.38–3.43; *p* < .001) and LAAV (HR = 1.06; 95% CI = 1.01–1.12; *p* = .029) were independently associated with AF recurrence in persistent AF, while no independent predictors could be identified in paroxysmal AF.

**Conclusion:**

The current study demonstrates that beyond left ventricular systolic dysfunction, LAA enlargement is associated with higher rate of AF recurrence after catheter ablation in persistent AF, but not in patients with paroxysmal AF.

**Abbreviations**
AAD: anti‐arrhythmic drug
AF: atrial fibrillation
BMI: body mass index
CAD: coronary artery disease
eGFR: estimated glomerular filtration rate
iLAV: body surface area‐indexed left atrial volume
LA: left atrium
LAA: left atrial appendage
LAAV: left atrial appendage volume
LVEF: left ventricular ejection fraction
PVI: pulmonary vein isolation
TIA: transient ischemic attack

## 1 INTRODUCTION

Atrial fibrillation (AF) is the most common sustained cardiac arrhythmia.^1^ It can lead to several health problems, such as heart failure, embolic events, and impaired quality of life. Moreover, it is associated with higher mortality rate.^2^, ^3^, ^4^ In case of drug‐resistant symptomatic AF, catheter ablation proved to be an effective solution for rhythm control.^5^ However, depending on the ablation strategy and the type of AF, success rates of catheter ablation after 1 year vary considerably from 60% to 90%.^5^, ^6^, ^7^ Appropriate patient selection for catheter ablation is essential as neither AF recurrence nor procedural complication rates are negligible.^8^ However, there are no consistently confirmed predictors of AF recurrence following catheter ablation in the literature.^9^, ^10^, ^11^, ^12^, ^13^, ^14^, ^15^, ^16^, ^17^, ^18^ It has been suggested that left atrial appendage (LAA) volume and function may be associated with the recurrence of AF/tachycardia in patients undergoing repeated ablation, the exact role of the LAA in the prediction of AF recurrence has not yet been resolved.^19^ The anatomy, including LAA volume, morphology and LAA orifice area can be accurately described using cardiac computed tomography (CT), and LAA function can be assessed by measuring LAA flow velocity using transesophageal echocardiography (TEE).

We aimed to study the role of LAA volume (LAAV), and function in the success of catheter ablation by type of AF.

## 2 METHODS

### 2.1 Study population

In our multimodality retrospective study, we included consecutive patients with symptomatic AF who underwent initial point‐by‐point radiofrequency catheter ablation at the Heart and Vascular Center of Semmelweis University, Budapest, Hungary between January 2014 and December 2017. All patients underwent preprocedural cardiac CT for the assessment of LA anatomy. LAA function was assessed with LAA flow velocity measured by TEE. Left ventricular ejection fraction (LVEF) was measured by transthoracic echocardiography using Biplane Simpson method. Exclusion criteria were age under 18 years, non‐diagnostic CT image quality, history of prior pulmonary vein isolation or heart surgery, and missing echocardiography data on LAA flow velocity. Due to the retrospective analysis of clinically acquired data, the institutional review board waived the need for written patient informed consent. The study was performed according to the Declaration of Helsinki and Institutional Guidelines.

### 2.2 Cardiac CT imaging

CT examinations were performed with a 256‐slice scanner (Brilliance iCT 256, Philips Healthcare, Best, The Netherlands) with prospective ECG‐triggered axial acquisition mode. 100–120 kV with 200–300 mAs tube current was used depending on patient anthropometrics. Image acquisition was performed with 128 × 0.625 mm detector collimation, and 270 ms gantry rotation time. For heart rate control 50–100 mg metoprolol was given orally and 5–20 mg intravenously, if necessary. In patients with a heart rate of <80/min, mid‐diastolic triggering was applied with 3%–5% padding (73%–83% of the R‐R interval), and in those with ≥80/min, systolic triggering was chosen (35%–45% of the R‐R interval) regardless of the presence of AF at the ECG during CT examination. In total 85–95 ml contrast material (Iomeron 400, Bracco Ltd., Milan, Italy) was injected with a flow rate of 4.5–5.5 ml/s via antecubital vein access using a four‐phasic injection protocol.^20^ Bolus tracking in the LA was used to obtain proper scan timing. All patients received 0.8 mg sublingual nitroglycerin between the native CT and CTA examinations. CT data sets were reconstructed with 0.8 mm slice thickness and 0.4 mm increments. The LA and LAA volumes were measured using a semiautomatic software tool (EP Planning, Philips IntelliSpace Portal, Philips Healthcare, Best, The Netherlands) and if needed the borders of LA and LAA, the orifices of the pulmonary veins and the level of the mitral valve were manually adjusted.

### 2.3 LAA flow velocity measurement

Maximum 24 h before ablation, all patients underwent TEE examination to exclude the presence of LAA thrombus. iE33 and Epiq 7C (Philips Medical System, Andover, MA) systems equipped with S5‐1 phased array and X7‐2t matrix TEE transducers were used. TEE was performed during conscious sedation. The LAA was imaged from 0°, 45°, 90°, and 135° views to detect spontaneous echo contrast, sludge or thrombus. Subsequently, a sample volume was placed at the middle portion of the LAA and the peak velocity of the outflow of the LAA was measured.

### 2.4 Catheter ablation procedure

The indications for AF ablation procedures were in accordance with the current guidelines.^1^, ^21^ Paroxysmal AF was defined as self‐terminating AF, in most cases within 48 hours. Some AF paroxysms continued up to 7 days.^21^ Persistent AF was defined as AF that lasts longer than 7 days.^21^ Conscious sedation was carried out in all cases with intravenous fentanyl, midazolam, and propofol. Basic vital parameters of the patients were monitored in all cases with non‐invasive blood pressure measurements every 10 min and continuous pulse oximetry. Femoral venous access was used for all procedures. Transseptal puncture was performed routinely with fluoroscopy guidance and pressure monitoring, while intracardiac echocardiography was also utilized in difficult cases. All ablations were performed with the support of an electroanatomical mapping system (either CARTO, Biosense Webster, Inc., Diamond Bar, CA, USA; or ENSITE, St. Jude Medical, Inc., MN, USA), and the LA fast anatomical map was fused with the cardiac CT images to guide ablation (temperature‐controlled mode, 43°C, 25–35 W, irrigated 4 mm tip catheter) in the majority of patients. Pulmonary vein isolation was performed in each patient. Moreover, in patients with long‐standing persistent AF, additional ablation lines were drawn at the discretion of the operating physician. All patients without complications were discharged the day after the procedure.

### 2.5 Follow‐up and definition of AF recurrence

After discharge, outpatient clinical follow‐up visits were scheduled at 3, 6, and 12 months after the procedure and at least once yearly thereafter. The follow‐up visits included clinical assessment of the patient and 24‐hour Holter ECG monitoring. Follow‐up data were registered in the structured reporting platform (Axis, Neumann Medical Ltd, Budapest, Hungary). Recurrence of AF was defined as the occurrence of atrial tachyarrhythmia that lasted for more than 30 s with or without symptoms.^1^, ^21^ AF recurrences during the first 90 days after catheter ablation were not included in order to exclude AF during this vulnerable „blanking period”, which might be only a temporary phenomenon due to the inflammation, maturation and healing of the ablated lesions.^22^, ^23^

### 2.6 Statistical analysis

Categorical variables are expressed as frequencies (percentages) and continuous values are expressed as mean ± SD. Normality of continuous parameters was tested with Shapiro–Wilk test. Tests for significance were conducted using Mann–Whitney‐Wilcoxon or Kruskal‐Wallis tests for continuous variables and Pearson's chi‐square or Fisher exact tests (in case of five or less observations) for categorical variables. The event‐free survival rate was estimated using Kaplan–Meier method and log‐rank test was applied for the comparisons between the various groups. Cumulative event rates were calculated with event or censoring times measured from the date of ablation. For patients who did not experience AF recurrence, their time‐to‐event measure was censored at the last follow‐up visit date.

To identify parameters associated with AF recurrence after catheter ablation, uni‐ and multivariate Cox proportional hazard regression model was executed. In the multivariate model, adjustment was made for age > 65 years, persistent AF, impaired eGFR (<60 ml/min/1.73 m^2^), body surface area‐indexed LA volume (iLAV) measured by CT, LVEF<50%), sex, obesity (defined as body mass index ≥ 30 kg/m^2^), hypertension, dyslipidemia, diabetes (Type I and II), prior stroke/transient ischemic attack, obstructive coronary artery disease, thyroid gland diseases (hypo‐ and hyperthyroidism), unsuccessful preablational anti‐arrhythmic drug (AAD) therapy (including sotalol, propafenone and amiodarone therapies), and LAAV. Thirty patients were randomly selected for interobserver agreement and analyzed using intraclass correlation coefficient (ICC). Relative risks were expressed as hazard ratios (HRs) with associated 95% confidence intervals (CIs). Two‐tailed *p* values smaller than .05 were considered significant. All statistical analyses were performed in R environment (version 3.6.1). Cox proportional hazard regression analysis was done using the 'survival' package (version 3.1–8). Kaplan–Meier curve and log‐rank test were performed using the 'survminer' (version 0.4.6).

## 3 RESULTS

### 3.1 Patient characteristics

A total of 561 patients were included in the current analysis. Mean age was 61.9 ± 10.2 years and 34.9% of the patients were female. Recurrence of AF was reported in 40.8% of the patients (34.6% in patients with paroxysmal and 53.5% in those with persistent AF). Median recurrence‐free time was 22.7 (9.3–43.1) months (21.8 [9.4–43.2] months in paroxysmal and 23.6 [9.0–42.6] months in persistent AF. An excellent interobserver agreement was obtained for both the iLAV (ICC = 0.99), and LAAV (ICC = 0.90) measurements. Correlation among LA and LAA parameters are reported in Figure S1. The proportion of individuals aged >65 years (40.7% vs. 49.3%; *p* = .046), female gender (30% vs. 41.9%; *p* = .005), persistent AF (25.9% vs. 43.2%; *p* < .001), and LVEF <50% (6.9% vs. 21.0%; *p* < .001) were significantly higher in patients with AF recurrence. Moreover, patients with AF recurrence had significantly higher iLAV (54.4 ± 19.3 ml/m^2^ vs. 61.8 ± 23.9 ml/m^2^; *p* < .001), LAAV (7.6 ± 3.2 ml vs. 8.8 ± 5.2 ml; *p* = .002) and LAA orifice area (387.6 ± 140.5 mm^2^ vs. 454.4 ± 167.7 mm^2^; *p* < .001). Anthropometric data, cardiovascular comorbidities, AAD therapy and imaging parameters are summarized in Table 1. Medications and procedural times are reported in Table S1.

**Table 1:** Patient characteristics

	All patients (*n* = 561)	No AF recurrence (*n* = 332)	AF recurrence (*n* = 229)	*p*
Anthropometric data and comorbidities				
Age > 65 years, *n* (%)	248 (44.2)	135 (40.7)	113 (49.3)	**.046**
Female, *n* (%)	561 (34.9)	100 (30.1)	96 (41.9)	**.005**
Persistent AF, *n* (%)	185 (33.0)	86 (25.9)	99 (43.2)	**<.001**
Obesity, *n* (%)	187 (33.3)	112 (33.7)	75 (32.8)	0.856
Hypertension, *n* (%)	411 (73.3)	238 (71.7)	173 (75.5)	0.333
Hyperlipidemia, *n* (%)	143 (25.5)	86 (25.9)	57 (24.9)	0.844
Diabetes, *n* (%)	82 (14.6)	50 (15.1)	32 (14.0)	0.808
Obstructive CAD, *n* (%)	51 (9.1)	28 (8.4)	23 (10.0)	0.552
Stroke/TIA, *n* (%)	43 (7.7)	27 (8.1)	16 (7.0)	0.747
Thyroid gland disease, *n* (%)	56 (10.0)	36 (10.8)	20 (8.7)	0.475
eGFR<60 ml/min/1.73 m^2^	138 (24.6)	83 (25.0)	55 (24.0)	0.842
Imaging parameters				
LVEF<50%, *n* (%)	71 (12.7)	23 (6.9)	48 (21.0)	**<.001**
iLAV (ml/m^2^)	57.4 ± 21.6	54.4 ± 19.3	61.8 ± 23.9	**<.001**
LAAV (ml)	8.1 ± 4.2	7.6 ± 3.2	8.8 ± 5.2	**.002**
LAA orifice area (mm^2^)	414.9 ± 155.6	387.6 ± 140.5	454.4 ± 167.7	**<.001**
LAA flow velocity (cm/s)	34.1 ± 13.0	34.1 ± 13.2	34.2 ± 12.9	0.965

Abbreviations: AAD, anti‐arrhythmic drug; AF, atrial fibrillation; CAD, coronary artery disease; eGFR, estimated glomerular filtration rate; iLAV, body surface area‐indexed left atrial volume; LAA, left atrial appendage; LAAV, left atrial volume; LVEF, left ventricular ejection fraction; TIA, transient ischemic attack.

**Figure 1:**
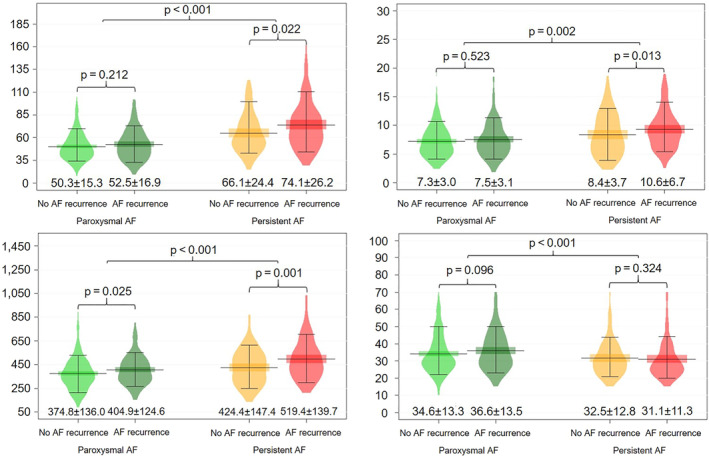
Comparison of LA and LAA parameters between patients with and without AF recurrence stratified by AF type. AF, atrial fibrillation; iLAV, body surface area‐indexed left atrial volume; LAA, left atrial appendage

We also examined the differences of the clinical and imaging parameters between patients with paroxysmal and persistent AF. Those patients with persistent AF had significantly higher proportion of age > 65 years (41.0% vs. 50.8%; *p* = .030), hypertension (67% vs. 85.9%; *p* < .001) and LVEF <50% (6.6% vs. 24.9%; *p* < .001). Regarding the CT parameters, we measured significantly higher iLAV (51.0 ± 15.9 ml/m^2^ vs. 70.4 ± 25.6 ml/m^2^; *p* < .001), LAAV (7.4 ± 3.0 ml vs. 9.5 ± 5.6 ml; *p* = .002), LAA orifice area (385.2 ± 132.8 mm^2^ vs. 475.2 ± 179.7 mm^2^; *p* < .001) and lower LAA flow velocity (35.3 ± 13.4 cm/s vs. 31.7 ± 12.0 cm/s; *p* < .001). Detailed data on the clinical and imaging parameters by AF type can be seen in Table 2 and Figure 1.

**Table 2:** Clinical characteristics by AF type

	Paroxysmal AF (*n* = 376)	Persistent AF (*n* = 185)	*p*
Age > 65 years, *n* (%)	154 (41.0)	94 (50.8)	**.030**
Female, *n* (%)	136 (36.2)	60 (32.4)	0.398
Obesity, *n* (%)	132 (35.1)	55 (29.7)	0.217
Hypertension, *n* (%)	252 (67.0)	159 (85.9)	**<.001**
Hyperlipidemia, *n* (%)	86 (22.9)	57 (30.8)	.050
Diabetes, *n* (%)	49 (13.0)	33 (17.8)	0.162
Obstructive CAD, *n* (%)	34 (9.0)	17 (9.2)	1.000
Stroke/TIA, *n* (%)	32 (8.5)	11 (5.9)	0.316
Thyroid gland disease, *n* (%)	46 (12.2)	10 (5.4)	.011
eGFR<60 ml/min/1.73 m^2^	100 (26.6)	38 (20.5)	0.144
Pre‐ablation AAD therapy, *n* (%)	196 (52.1)	87 (47.0)	0.281
LVEF<50%, *n* (%)	25 (6.6)	46 (24.9)	**<.001**

Abbreviations: AAD, anti‐arrhythmic drug; AF, atrial fibrillation; CAD, coronary artery disease; eGFR, estimated glomerular filtration rate; LVEF, left ventricular ejection fraction; TIA, transient ischemic attack.

### 3.2 Predictors of AF recurrence

Significantly higher iLAV and LAAV values were measured in patients with persistent AF recurrences, and larger LAA orifice area values were measured both in paroxysmal and persistent recurrences, as reported in Figure 1. To explore the associations between the various examined parameters and AF recurrence, Cox proportional hazards regression analyses were performed, as stratified by AF type. In the univariate analysis, female sex (HR = 1.43; 95% CI = 1.01–2.02; *p* = .043) was significantly associated with AF recurrence in patients with paroxysmal AF, while in those with persistent AF LVEF <50% (HR = 2.07; 95% CI = 1.36–3.14; *p* < .001), iLAV (HR = 1.01; 95% CI = 1.00–1.02; *p* = .027), LAAV (HR = 1.07; 95% CI = 1.03–1.10; *p* < .001) and LAA orifice area (HR = 1.02; 95% CI = 1.00–1.03 per 10 mm^2^; *p* = .005) showed an association with AF recurrence.

**Figure 2:**
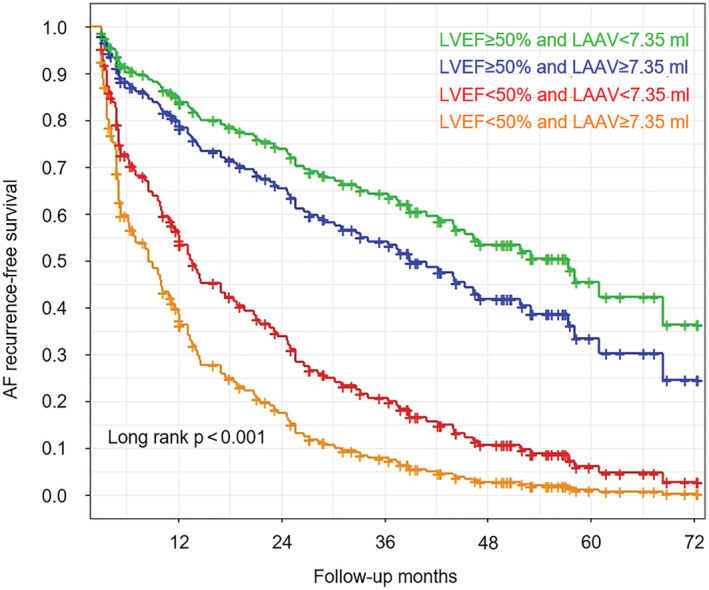
Adjusted AF recurrence‐free survival according to LVEF and LAAV in patients with persistent AF. The lines represent the marginal means of the estimated AF recurrence‐free survival. Marginal means were estimated from the Cox proportional hazard regression model adjusted for age > 65 years, impaired eGFR, iLAV, sex, obesity, hypertension, dyslipidemia, diabetes, prior stroke/TIA, obstructive CAD, thyroid gland diseases and unsuccessful preablational AAD therapy. Median of LAAV was used as cut‐off value. AF, atrial fibrillation; LAAV, left atrial appendage volume; LVEF, left ventricular ejection fraction

After adjustment LVEF <50% (HR = 2.17; 95% CI = 1.38–3.43; *p* < .001) and LAAV (HR = 1.06; 95% CI = 1.01–1.12; *p* = .029) remained a significant predictor of AF recurrence in patients with persistent AF, while in paroxysmal AF no independent predictors could be identified in the multivariate analysis. Detailed results of the uni‐ and multivariate Cox regression analyses are reported in Table 3. Kaplan–Meier curves of AF recurrence‐free survival in persistent AF stratified by LVEF and LAAV can be seen in Figure 2, Figures S2 and S3.

**Table 3:** Associates of AF recurrence in patients with paroxysmal AF

	Paroxysmal AF						Persistent AF					
	Unadjusted analysis			Adjusted analysis			Unadjusted analysis			Adjusted analysis		
	HR	95% CI	*p*	HR	95% CI	*p*	HR	95% CI	*p*	HR	95% CI	*p*
Age > 65 years, *n* (%)	1.01	0.71–1.42	0.967	1.02	0.69–1.51	0.916	1.08	0.72–1.60	0.722	0.90	0.55–1.47	0.684
Female, *n* (%)	**1.43**	**1.01–2.02**	**.043**	1.42	0.96–2.11	.078	1.33	0.89–2.00	0.163	1.35	0.82–2.22	0.233
Obesity, *n* (%)	1.08	0.75–1.54	0.685	1.09	0.75–1.59	0.651	1.07	0.69–1.66	0.764	1.06	0.64–1.77	0.811
Hypertension, *n* (%)	0.96	0.66–1.39	0.816	0.91	0.60–1.37	0.648	1.03	0.59–1.78	0.921	1.13	0.59–2.13	0.717
Hyperlipidemia, *n* (%)	0.82	0.54–1.24	0.338	0.86	0.56–1.32	0.481	0.87	0.57–1.35	0.541	0.82	0.50–1.37	0.452
Diabetes, *n* (%)	0.77	0.45–1.33	0.353	0.79	0.45–1.39	0.481	0.92	0.54–1.55	0.745	1.10	0.61–1.97	0.752
Obstructive CAD, *n* (%)	1.08	0.62–1.89	0.781	1.28	0.70–2.33	0.426	0.98	0.49–1.95	0.961	1.64	0.71–3.75	0.246
Stroke/TIA, *n* (%)	0.88	0.47–1.62	0.673	0.96	0.50–1.82	0.890	0.77	0.31–1.89	0.567	0.43	0.16–1.15	.091
Thyroid gland disease, *n* (%)	0.94	0.55–1.58	0.806	0.85	0.48–1.49	0.565	0.87	0.32–2.37	0.787	0.64	0.22–1.89	0.418
eGFR<60 ml/min/1.73 m^2^	1.38	0.93–2.06	0.111	1.30	0.85–2.00	0.229	0.89	0.55–1.41	0.610	0.87	0.52–1.46	0.596
Pre‐ablation AAD therapy, *n* (%)	0.96	0.68–1.36	0.836	1.00	0.70–1.43	0.983	0.97	0.65–1.44	0.868	0.89	0.57–1.39	0.598
LVEF<50%, *n* (%)	1.67	0.96–2.91	.069	1.42	0.80–2.52	0.232	**2.07**	**1.36–3.14**	**<.001**	**2.17**	**1.38–3.43**	**<.001**
iLAV (ml/m^2^)	1.01	0.99–1.02	.098	1.01	0.99–1.02	0.330	**1.01**	**1.00–1.02**	**.027**	1.00	0.99–1.01	0.549
LAAV (ml)	1.03	0.97–1.09	0.325	1.00	0.93–1.06	0.889	**1.07**	**1.03–1.10**	**<.001**	**1.06**	**1.01–1.12**	**.029**
LAA orifice area (mm^2^), per 10 mm^2^	1.01	1.00–1.03	.034	1.00	1.00–1.00	0.226	1.02	1.00–1.03	0.005	1.00	1.00–1.01	0.717
LAA flow velocity (cm/s)	1.00	0.99–1.03	0.352	1.01	1.00–1.02	.079	1.00	0.98–1.02	0.919	1.00	0.98–1.02	0.812

Abbreviations: AAD, anti‐arrhythmic drug; AF, atrial fibrillation; CAD, coronary artery disease; eGFR, estimated glomerular filtration rate; iLAV, body surface area‐indexed left atrial volume; LAA, left atrial appendage; LAAV, left atrial volume; LVEF, left ventricular ejection fraction; TIA, transient ischemic attack.

Statistically significant *p*‐values were marked bold.

## 4 DISCUSSION

We demonstrated that beyond impaired LVEF, a larger LAAV is an independent predictor of AF recurrence after catheter ablation in patients with persistent AF. Interestingly, this association was not present in patients with paroxysmal AF.

AF is a complex disease with incompletely understood mechanisms. Although significant progress has been made in the last two decades, the efficacy of ablation therapy remains suboptimal, particularly in persistent AF. One‐year success rate of catheter ablation varies between 60% and 90%.^5^, ^6^, ^7^ Previous studies have shown that the majority of AF recurrence occurred in the first 2 years after catheter ablation.^24^ So far, persistent AF, LA enlargement, hypertension, diabetes mellitus, aging, obesity, heart failure, chronic renal insufficiency and preprocedural amiodarone failure have been reported as independent predictors of AF recurrence.^1^, ^21^, ^24^, ^25^, ^26^, ^27^ However, the data are controversial and the conclusions of previous studies are inconsistent. Several studies aimed to investigate the role of different scoring systems in the prediction of rhythm outcomes after AF ablation. While the HATCH score was found to have no value in the prediction of AF recurrence after catheter ablation_,_ R_2_CHADS_2_ and CHA_2_DS_2_‐VASc scores were associated with rhythm outcomes.^28^, ^29^ Since APPLE score proved to be superior to the CHA_2_DS_2_‐VASc score for the prediction of rhythm outcome after catheter ablation, we incorporated its factors into our multivariable models.^30^ Due to inconsistent definition of recurrence, estimation of the AF ablation success is challenging.^1^, ^21^ Current guidelines of the European Society of Cardiology define AF recurrence as the occurrence of atrial tachyarrhythmia that last for more than 30 s.^1^, ^21^ In the current study, we have also applied this definition. Moreover, previous studies have reported a wide range of recurrent AF duration time following various ablation strategies. In our study, we included AF patients who underwent point‐by‐point catheter ablation procedure after 2014 in order to provide more useful information to the current clinical practice. Moreover, since efficacy of radiofrequency catheter ablation varies greatly between paroxysmal and persistent AF, we analyzed the outcomes separately by type of AF. Since radiofrequency catheter ablation procedure became more widely performed, clinical studies regarding the long‐term effectiveness are important, especially in patients with persistent AF. In our study population the recurrence rate after point‐by‐point catheter ablation was 53.5% in persistent AF and 34.6 in paroxysmal AF after a single procedure. These findings suggest that catheter ablation in patients with persistent AF should be chosen very cautiously due to the low success rate and non‐negligible complication rate. In the present study, left ventricular systolic dysfunction and higher LAAV were identified as significant predictors of AF recurrence in patients with persistent AF who underwent point‐by‐point catheter ablation.

Di Biase et al have reported that LAA appears to be responsible for recurrence of AF/tachycardia in at least 27% of patients undergoing repeated ablation, especially in persistent AF cases.^19^ Moreover, electric isolation of the LAA was associated with an improved AF‐free/recurrence‐free survival.^31^ Despite the increasing evidence of the role of LAA in triggering atrial arrhythmias, the literature is scarce regarding the contributing factors. Previous smaller studies including both paroxysmal and persistent AF patients undergoing catheter ablation have shown that larger LAAV is associated with a higher risk of AF recurrence.^32^, ^33^ However, in these studies patients were followed up for 12 and 24 months, respectively, while our analysis studied AF recurrences during a longer follow‐up period. As compared to their results, in our study population AF recurrence rate was 18% versus 32% at 12 months and 29% versus 33% at 24 months, respectively. Moreover, the above‐mentioned studies included 52 and 62 patients as compared to 561 individuals in our analysis. Both studies were in line with our findings regarding greater LAAV values in patients with AF recurrence. The LAA is known to be more compliant than the LA, and therefore may play an important role in the modulation of LA pressure and LAAV measurement could be a reliable tool in determining the structural and functional conditions of LA from the early stage of AF.^34^ In line with these findings, our results also suggest that LAAV may be a surrogate of increased LAA arrhythmogenicity. LAA has a complex anatomy and LAA enlargement might result in longer activation pathways and development of re‐entry through interstitial fibrosis.^35^ Previous studies have shown that LAA remodeling (dilatation, stretching, reduction of pectinate muscles, endocardial fibroelastosis) can occur in chronic AF.^36^, ^37^ This might explain that larger LAAV was associated with persistent, but not with paroxysmal AF in our study.

The current study has several limitations. First, it was a single‐center, retrospective study, therefore the results need further validation in prospective, multicenter studies. Second, even if patients were closely followed‐up with regular clinical visits, ECG and Holter monitoring, asymptomatic AF episodes might have happened between two visits. Moreover, ablation strategy was not uniform in all subjects, as additional ablations were permitted beyond PVI if operator decided to draw additional lines. However, our analysis includes a large number of patients with a long follow‐up period. In addition, we included patients only who underwent point‐by‐point radiofrequency catheter ablation, which ensures homogenous cohort in terms of procedure technique. Furthermore, in order to exclude AF recurrences during the “blanking period” when arrhythmias might be caused by postprocedural inflammation and maturation, cases during the first 90 days were not included in our analysis. Another strength of our study is that all cardiac CT was performed by a 256‐slice machine, which allows more precise imaging and measurement of LA and LAA volumes.

## 5 CONCLUSION

Despite advances in catheter ablation techniques, AF recurrence rate is relatively high. The current study demonstrates that beyond left ventricular systolic dysfunction, LAA enlargement is an independent predictor of AF recurrence after catheter ablation in persistent AF. Our results suggest that preprocedural assessment of LVEF and LAAV might contribute to optimal patient selection and aid to improve long‐term results of ablation procedures in patients with persistent AF.

## CONFLICT OF INTEREST

The authors have no conflicts to disclose.

## Supporting information


**Figure S1** Spearman correlation coefficients for the relationship among iLAV, LAAV, LAA orifice area and LAA flow velocity.
Abbreviations: iLAV = body surface area‐indexed left atrial volume; LAA = left atrial appendage; LAAV = left atrial appendage volume.

**Figure S2** AF recurrence‐free survival according to LAAV in patients with persistent AF
Median of LAAV was used as cut‐off value.
Abbreviations: LAAV = Left atrial appendage.

**Figure S3** AF recurrence‐free survival according to LAAV and LVEF in patients with persistent AF
Median of LAAV was used as cut‐off value.
Abbreviations: LAAV = Left atrial appendage, LVEF = Left ventricular ejection fraction.

**Table S1** Abbreviations: LA = left atrium; LMWH = light molecular weight heparin; NOAC = non‐Vitamin K antagonist oral anticoagulation; OAC = oral anticoagulant.

## Data Availability

The data underlying this article will be available on reasonable request to the corresponding author.

## References

[1] Calkins H , Hindricks G , Cappato R , et al. 2017 HRS/EHRA/ECAS/APHRS/SOLAECE expert consensus statement on catheter and surgical ablation of atrial fibrillation: executive summary. J Arrhythm. 2017;33(5):369‐409. doi:10.1016/j.joa.2017.08.001 29021841 PMC5634725

[2] Dorian P , Jung W , Newman D , et al. The impairment of health‐related quality of life in patients with intermittent atrial fibrillation: implications for the assessment of investigational therapy. J Am Coll Cardiol. 2000;36(4):1303‐1309. doi:10.1016/s0735-1097(00)00886-x 11028487

[3] Wang TJ , Larson MG , Levy D , et al. Temporal relations of atrial fibrillation and congestive heart failure and their joint influence on mortality: the Framingham heart study. Circulation. 2003;107(23):2920‐2925. doi:10.1161/01.CIR.0000072767.89944.6E 12771006

[4] Wolf PA , Abbott RD , Kannel WB . Atrial fibrillation as an independent risk factor for stroke: the Framingham study. Stroke. 1991;22(8):983‐988. doi:10.1161/01.str.22.8.983 1866765

[5] Oral H , Pappone C , Chugh A , et al. Circumferential pulmonary‐vein ablation for chronic atrial fibrillation. N Engl J Med. 2006;354(9):934‐941. doi:10.1056/NEJMoa050955 16510747

[6] Cappato R , Calkins H , Chen SA , et al. Worldwide survey on the methods, efficacy, and safety of catheter ablation for human atrial fibrillation. Circulation. 2005;111(9):1100‐1105. doi:10.1161/01.CIR.0000157153.30978.67 15723973

[7] Wazni OM , Marrouche NF , Martin DO , et al. Radiofrequency ablation vs antiarrhythmic drugs as first‐line treatment of symptomatic atrial fibrillation: a randomized trial. JAMA. 2005;293(21):2634‐2640. doi:10.1001/jama.293.21.2634 15928285

[8] Szegedi N , Szeplaki G , Herczeg S , et al. Repeat procedure is a new independent predictor of complications of atrial fibrillation ablation. Europace. 2019;21(5):732‐737. doi:10.1093/europace/euy326 30689857 PMC6479506

[9] Bhargava M , Di Biase L , Mohanty P , et al. Impact of type of atrial fibrillation and repeat catheter ablation on long‐term freedom from atrial fibrillation: results from a multicenter study. Heart Rhythm. 2009;6(10):1403‐1412. doi:10.1016/j.hrthm.2009.06.014 19716348

[10] Gerstenfeld EP , Sauer W , Callans DJ , et al. Predictors of success after selective pulmonary vein isolation of arrhythmogenic pulmonary veins for treatment of atrial fibrillation. Heart Rhythm. 2006;3(2):165‐170. doi:10.1016/j.hrthm.2005.10.016 16443531

[11] Helms AS , West JJ , Patel A , et al. Relation of left atrial volume from three‐dimensional computed tomography to atrial fibrillation recurrence following ablation. Am J Cardiol. 2009;103(7):989‐993. doi:10.1016/j.amjcard.2008.12.021 19327428

[12] Li C , Ding X , Zhang J , Zhou C , Chen Y , Rao L . Does the E/e' index predict the maintenance of sinus rhythm after catheter ablation of atrial fibrillation? Echocardiography. 2010;27(6):630‐636. doi:10.1111/j.1540-8175.2009.01123.x 20412266

[13] Lim TW , Jassal IS , Ross DL , Thomas SP . Medium‐term efficacy of segmental ostial pulmonary vein isolation for the treatment of permanent and persistent atrial fibrillation. Pacing Clin Electrophysiol. 2006;29(4):374‐379. doi:10.1111/j.1540-8159.2006.00356.x 16650265

[14] Park SM , Kim YH , Choi JI , Pak HN , Kim YH , Shim WJ . Left atrial electromechanical conduction time can predict six‐month maintenance of sinus rhythm after electrical cardioversion in persistent atrial fibrillation by Doppler tissue echocardiography. J Am Soc Echocardiogr. 2010;23(3):309‐314. doi:10.1016/j.echo.2009.12.019 20138472

[15] Tilz RR , Rillig A , Thum AM , et al. Catheter ablation of long‐standing persistent atrial fibrillation: 5‐year outcomes of the Hamburg sequential ablation strategy. J Am Coll Cardiol. 2012;60(19):1921‐1929. doi:10.1016/j.jacc.2012.04.060 23062545

[16] Vasamreddy CR , Lickfett L , Jayam VK , et al. Predictors of recurrence following catheter ablation of atrial fibrillation using an irrigated‐tip ablation catheter. J Cardiovasc Electrophysiol. 2004;15(6):692‐697. doi:10.1046/j.1540-8167.2004.03538.x 15175066

[17] Yasuda T , Kumagai K , Ogawa M , et al. Predictors of successful catheter ablation for atrial fibrillation using the pulmonary vein isolation technique. J Cardiol. 2004;44(2):53‐58. 15373237

[18] Berruezo A , Tamborero D , Mont L , et al. Pre‐procedural predictors of atrial fibrillation recurrence after circumferential pulmonary vein ablation. Eur Heart J. 2007;28(7):836‐841. doi:10.1093/eurheartj/ehm027 17395676

[19] Di Biase L , Burkhardt JD , Mohanty P , et al. Left atrial appendage: an underrecognized trigger site of atrial fibrillation. Circulation. 2010;122(2):109‐118. doi:10.1161/CIRCULATIONAHA.109.928903 20606120

[20] Karady J , Panajotu A , Kolossvary M , et al. The effect of four‐phasic versus three‐phasic contrast media injection protocols on extravasation rate in coronary CT angiography: a randomized controlled trial. Eur Radiol. 2017;27(11):4538‐4543. doi:10.1007/s00330-017-4866-0 28540480 PMC5635079

[21] Kirchhof P , Benussi S , Kotecha D , et al. 2016 ESC guidelines for the management of atrial fibrillation developed in collaboration with EACTS. Europace. 2016;18(11):1609‐1678. doi:10.1093/europace/euw295 27567465

[22] Deftereos S , Giannopoulos G , Kossyvakis C , et al. Colchicine for prevention of early atrial fibrillation recurrence after pulmonary vein isolation: a randomized controlled study. J Am Coll Cardiol Oct 30. 2012;60(18):1790‐1796. doi:10.1016/j.jacc.2012.07.031 23040570

[23] European Heart Rhythm A, European Cardiac Arrhythmia S, American College of C et al. HRS/EHRA/ECAS expert consensus statement on catheter and surgical ablation of atrial fibrillation: recommendations for personnel, policy, procedures and follow‐up. A report of the Heart Rhythm Society (HRS) task force on catheter and surgical ablation of atrial fibrillation. Heart Rhythm. 2007;4(6):816‐861. doi:10.1016/j.hrthm.2007.04.005 17556213

[24] Mujovic NM , Marinkovic MM , Potpara TS , Geller L . Catheter ablation of lone atrial fibrillation. Curr Pharm des. 2015;21(5):591‐612. doi:10.2174/1381612820666140825144226 25175086

[25] Hussein AA , Saliba WI , Martin DO , et al. Natural history and long‐term outcomes of ablated atrial fibrillation. Circ Arrhythm Electrophysiol. 2011;4(3):271‐278. doi:10.1161/CIRCEP.111.962100 21493959

[26] Mohanty S , Mohanty P , Di Biase L , et al. Impact of metabolic syndrome on procedural outcomes in patients with atrial fibrillation undergoing catheter ablation. J Am Coll Cardiol. 2012;59(14):1295‐1301. doi:10.1016/j.jacc.2011.11.051 22464257

[27] Shah AN , Mittal S , Sichrovsky TC , et al. Long‐term outcome following successful pulmonary vein isolation: pattern and prediction of very late recurrence. J Cardiovasc Electrophysiol. 2008;19(7):661‐667. doi:10.1111/j.1540-8167.2008.01101.x 18284502

[28] Kornej J , Hindricks G , Kosiuk J , et al. Comparison of CHADS2, R2CHADS2, and CHA2DS2‐VASc scores for the prediction of rhythm outcomes after catheter ablation of atrial fibrillation: the Leipzig heart center AF ablation registry. Circ Arrhythm Electrophysiol. 2014;7(2):281‐287. doi:10.1161/CIRCEP.113.001182 24610790

[29] Tang RB , Dong JZ , Long DY , et al. Efficacy of catheter ablation of atrial fibrillation beyond HATCH score. Chin Med J (Engl). 2012;125(19):3425‐3429. 23044300

[30] Kornej J , Hindricks G , Shoemaker MB , et al. The APPLE score: a novel and simple score for the prediction of rhythm outcomes after catheter ablation of atrial fibrillation. Clin Res Cardiol. 2015;104(10):871‐876. doi:10.1007/s00392-015-0856-x 25876528 PMC4726453

[31] Di Biase L , Burkhardt JD , Mohanty P , et al. Left atrial appendage isolation in patients with longstanding persistent AF undergoing catheter ablation: BELIEF trial. J Am Coll Cardiol. 2016;68(18):1929‐1940. doi:10.1016/j.jacc.2016.07.770 27788847

[32] Zheng GA , Lin CY , Weng L , Chen JD . Left atrial appendage volume is a valuable predictor of atrial fibrillation recurrence after radiofrequency catheter ablation. Zhonghua Xin Xue Guan Bing Za Zhi. 2017;45(11):924‐929. doi:10.3760/cma.j.issn.0253-3758.2017.11.006 29166717

[33] Pinto Teixeira P , Martins Oliveira M , Ramos R , et al. Left atrial appendage volume as a new predictor of atrial fibrillation recurrence after catheter ablation. J Interv Card Electrophysiol. 2017;49(2):165‐171. doi:10.1007/s10840-017-0256-4 28567579

[34] Tabata T , Oki T , Yamada H , et al. Role of left atrial appendage in left atrial reservoir function as evaluated by left atrial appendage clamping during cardiac surgery. Am J Cardiol. 1998;81(3):327‐332. doi:10.1016/s0002-9149(97)00903-x 9468076

[35] Krul SP , Berger WR , Smit NW , et al. Atrial fibrosis and conduction slowing in the left atrial appendage of patients undergoing thoracoscopic surgical pulmonary vein isolation for atrial fibrillation. Circ Arrhythm Electrophysiol. 2015;8(2):288‐295. doi:10.1161/CIRCEP.114.001752 25673630

[36] Shirani J , Alaeddini J . Structural remodeling of the left atrial appendage in patients with chronic non‐valvular atrial fibrillation: implications for thrombus formation, systemic embolism, and assessment by transesophageal echocardiography. Cardiovasc Pathol. 2000;9(2):95‐101. doi:10.1016/s1054-8807(00)00030-2 10867359

[37] Rubin DN , Katz SE , Riley MF , Douglas PS , Manning WJ . Evaluation of left atrial appendage anatomy and function in recent‐onset atrial fibrillation by transesophageal echocardiography. Am J Cardiol. 1996;78(7):774‐778. doi:10.1016/s0002-9149(96)00419-5 8857481

