# Supplementary figures and images for: Left atrial appendage size is a marker of atrial fibrillation recurrence after radiofrequency catheter ablation in patients with persistent atrial fibrillation
Source: Clin Cardiol. 2021 Nov 19;45(3):273–81. doi: 10.1002/clc.23748 (PMC8922535; doi:10.1002/clc.23748)

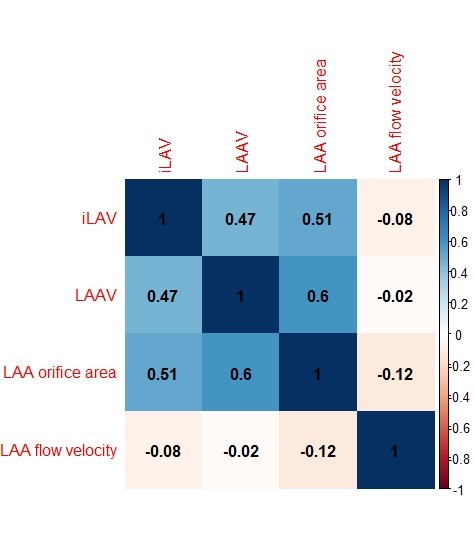

Supplement: Supplementary file 1 — Figure S1 Spearman correlation coefficients for the relationship among iLAV, LAAV, LAA orifice area and LAA flow velocity. Abbreviations: iLAV = body surface area‐indexed left atrial volume; LAA = left atrial appendage; LAAV = left atrial appendage volume. [file CLC-45-273-s004.jpg]

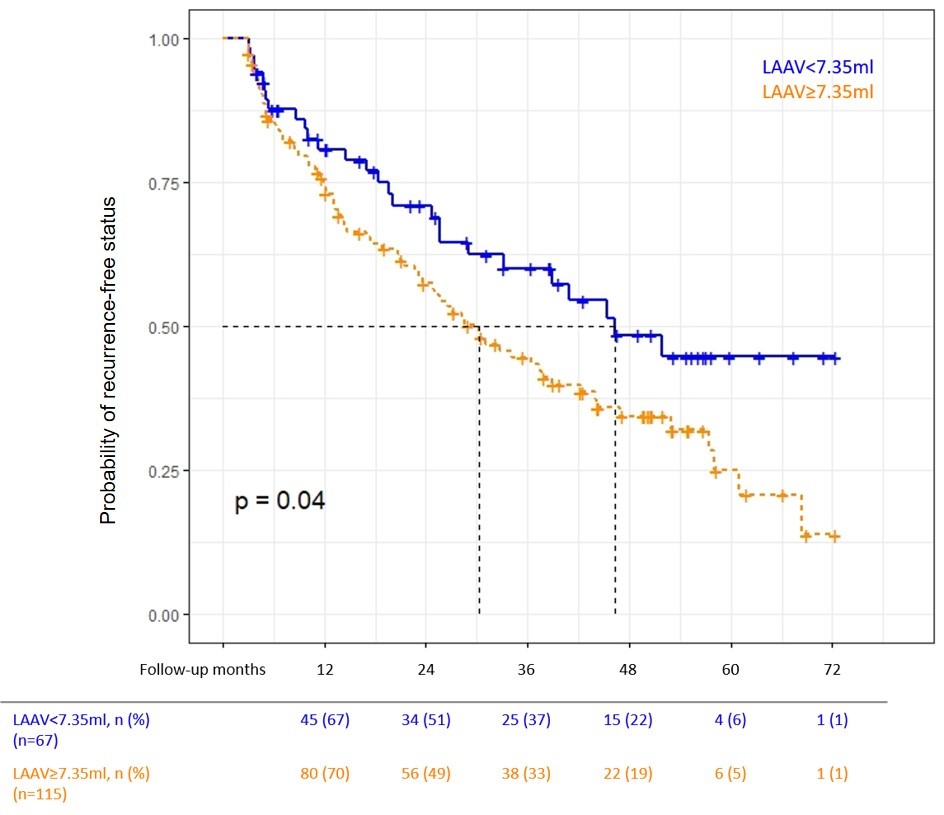

Supplement: Supplementary file 2 — Figure S2 AF recurrence‐free survival according to LAAV in patients with persistent AF Median of LAAV was used as cut‐off value. Abbreviations: LAAV = Left atrial appendage. [file CLC-45-273-s002.jpg]

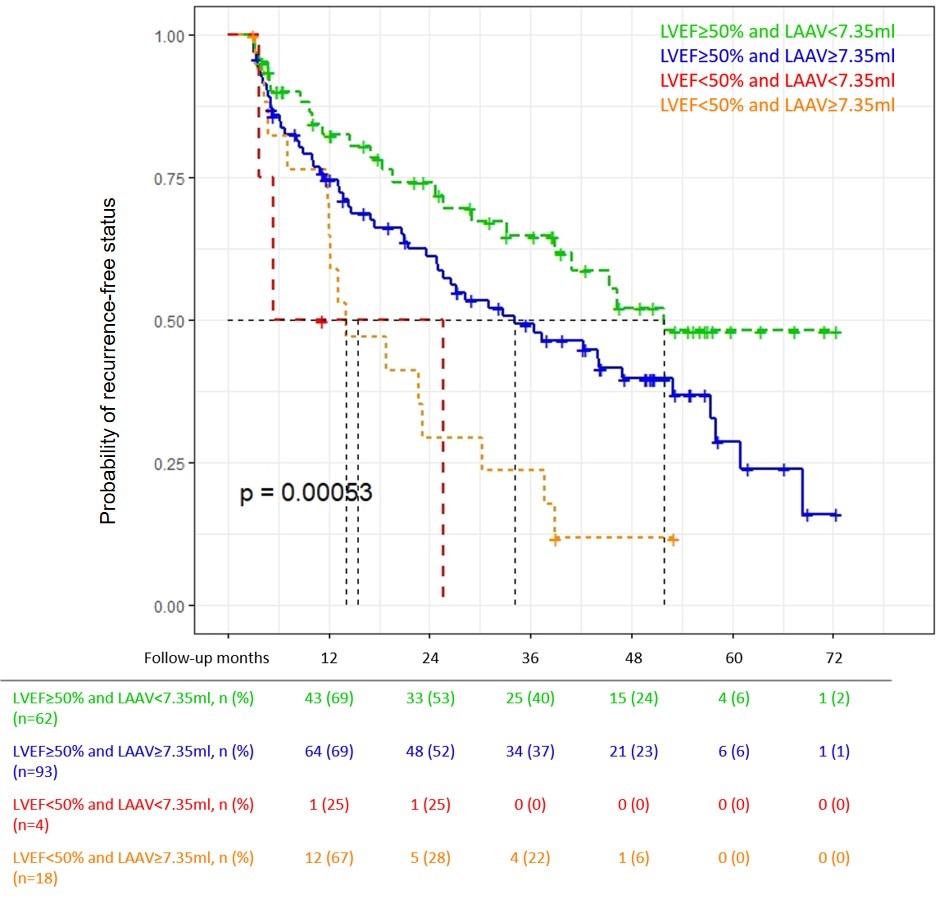

Supplement: Supplementary file 3 — Figure S3 AF recurrence‐free survival according to LAAV and LVEF in patients with persistent AF Median of LAAV was used as cut‐off value. Abbreviations: LAAV = Left atrial appendage, LVEF = Left ventricular ejection fraction. [file CLC-45-273-s003.jpg]
