# Supplementary material for: Left atrial appendage size is a marker of atrial fibrillation recurrence after radiofrequency catheter ablation in patients with persistent atrial fibrillation
Source: Clin Cardiol. 2021 Nov 19;45(3):273–81. doi: 10.1002/clc.23748 (PMC8922535; doi:10.1002/clc.23748)
Supplement: Supplementary file 4 — Table S1 Abbreviations: LA = left atrium; LMWH = light molecular weight heparin; NOAC = non‐Vitamin K antagonist oral anticoagulation; OAC = oral anticoagulant. [file CLC-45-273-s001.docx]

Supplementary table 1.

| **Medications** | |
| --- | --- |
| NOAC, n (%) | 312 (55.6) |
| OAC, n (%) | 237 (42.2) |
| Syncumar, n (%) | 74 (13.2) |
| Warfarin, n (%) | 121 (21.6) |
| Dabigatran, n (%) | 113 (20.1) |
| Rivaroxaban, n (%) | 100 (17.8) |
| LMWH, n (%) | 251 (44.7) |
| Antiplatelet therapy, n (%) | 13 (2.3) |
| Apixaban, n (%) | 24 (4.3) |
| AAD therapy, n (%) | 283 (50.4) |
| Sotalol, n (%) | 47 (8.4) |
| Amiodarone, n (%) | 139 (24.8) |
| Propafenon, n (%) | 97 (17.3) |
| **Procedural times** | |
| Procedure time (min) | 94.7±28.1 |
| LA time (min) | 64.8±23.5 |
| Fluoroscopy time (min) | 9.7±6.5 |

Abbreviations: LA = left atrium; LMWH = light molecular weight heparin; NOAC = non-Vitamin K antagonist oral anticoagulation; OAC = oral anticoagulant.
